# Assessing *EGFR*‐mutated NSCLC with bone metastasis: Clinical features and optimal treatment strategy

**DOI:** 10.1002/cam4.7152

**Published:** 2024-03-29

**Authors:** Wei‐Chun Chen, Wen‐Chien Cheng, Chieh‐Lung Chen, Wei‐Chih Liao, Chia‐Hung Chen, Hung‐Jen Chen, Chih‐Yen Tu, Chi‐Chen Lin, Te‐Chun Hsia

**Affiliations:** ^1^ Division of Pulmonary and Critical Care, Department of Internal Medicine China Medical University Hospital Taichung Taiwan; ^2^ School of Medicine, College of Medicine, China Medical University Taichung Taiwan; ^3^ Department of Life Science National Chung Hsing University Taichung Taiwan; ^4^ National Chung Hsing University Taichung Taiwan; ^5^ Rong Hsing Research Center for Translational Medicine National Chung Hsing University Taichung Taiwan; ^6^ Institute of Biomedical Science, The iEGG and Animal Biotechnology Center National Chung‐Hsing University Taichung Taiwan; ^7^ Department of Medical Research China Medical University Hospital Taichung Taiwan; ^8^ Department of Medical Research Taichung Veterans General Hospital Taichung Taiwan; ^9^ Department of Pharmacology College of Medicine, Kaohsiung Medical University Kaohsiung Taiwan

**Keywords:** antiangiogenesis, bone metastasis, chemotherapy, denosumab, epidermal growth factor receptor, tyrosine kinase inhibitor

## Abstract

**Background:**

This study aimed to examine the clinical characteristics of bone metastasis (BoM) in patients with non‐small cell lung cancer (NSCLC) who have an epidermal growth factor receptor (EGFR) mutation and to identify the most effective treatment strategy using EGFR–tyrosine kinase inhibitors (TKIs).

**Methods:**

The study included patients with stage IV EGFR‐mutated NSCLC who were receiving first‐line treatment with EGFR–TKIs between January 2014 and December 2020. These patients were divided into two groups based on the presence or absence of BoM at the time of initial diagnosis. The BoM group was further subdivided based on whether they received denosumab or not.

**Results:**

The final analysis included 247 patients. Those with BoM at initial diagnosis had shorter progression‐free survival (12.6 vs. 10.5 months, *p* = 0.002) and overall survival (OS) (49.7 vs. 30.9 months, *p* = 0.002) compared to those without BoM. There was a difference in the location of metastatic sites between the two groups, with a higher incidence of extrathoracic metastasis in the BoM group (*p* < 0.001). The incidence of T790M was higher in patients with BoM than in those without (47.4% vs. 33.9%, *p* = 0.042). Multivariate Cox regression analysis revealed that sequential osimertinib treatment and the addition of antiangiogenic therapy (AAT) and denosumab therapy improved OS in patients with BoM.

**Conclusions:**

The presence of BoM is a negative prognostic factor for NSCLC patients with an EGFR mutation, possibly due to the presence of extrathoracic metastases. However, adding AAT and denosumab, along with sequential osimertinib, to the treatment regimen for patients with BoM can improve survival outcomes.

## 1 INTRODUCTION

In recent decades, lung cancer has been the world's leading cause of cancer death, despite the use of more effective treatments such as chemotherapy, immunotherapy, and targeted therapy.^1^ About 15%–20% of non‐Asian patients and 40%–60% of Asian patients diagnosed with non‐small cell lung cancer (NSCLC) have mutations in the epidermal growth factor receptor (EGFR).^2^, ^3^ Patients with advanced NSCLC with classic EGFR mutations (exon 19 deletion or L858R mutation) are treated as first‐line standard drugs with EGFR–tyrosine kinase inhibitors (TKIs). This includes the first‐generation EGFR–TKIs like gefitinib and erlotinib, second‐generation EGFR–TKI such as afatinib and dacomitinib, as well as the third‐generation EGFR–TKI, osimertinib. Initially approved for patients with acquired T790M mutations after failure on first‐ or second‐generation EGFR–TKIs, osimertinib was later assessed as a first‐line treatment due to its effectiveness against classic EGFR mutations and T790M mutations.^4^ Significant proportions of patients with EGFR mutation‐positive NSCLC develop bone metastases (BoM), and about 40% develop it during their disease course. BoM is associated with a greater incidence of skeletal complications, lower quality of life, and a decrease in overall survival (OS).^5^, ^6^ According to previous studies, it has been shown that BoM is an independent negative prognostic factor for patients with EGFR‐positive NSCLC.^7^ BoM is not only linked to skeletal complications but also to multi‐organ metastases, including brain and liver metastases, which are indicators of poor prognosis in patients with EGFR‐mutated NSCLC.^8^ Therefore, identification of the optimal treatment strategy for these patients is urgently needed.

The available treatment options for BoM in patients with NSCLC include radiotherapy, denosumab, and zoledronic acid.^9^ Denosumab is a fully human monoclonal antibody with high affinity and specificity for the receptor activator of nuclear factor kappa‐B ligand (RANKL). It acts as an endogenous inhibitor, similar to osteoprotegerin.^10^, ^11^, ^12^ RANKL activates RANK, a member of the TNF receptor family, promoting the maturation of preosteoclasts into osteoclasts.^13^, ^14^ Denosumab inhibits the interaction between RANK and RANKL, thus preventing the activation of osteoclasts and halting the vicious cycle of bone degradation.^10^, ^15^, ^16^ In a mouse model of metastatic NSCLC, suppression of RANKL reduced the burden of skeletal tumors and increased survival.^17^ A phase III study found that in patients with metastatic lung cancer, denosumab was associated with better OS compared to zoledronic acid.^9^ Additionally, a retrospective study showed that denosumab improved OS in non‐squamous cell lung cancer, with a median survival of 21.4 months, compared to 12.7 months for zoledronic acid.^11^

Limited research has been conducted on improving the prognosis of NSCLC patients with BoM who have EGFR mutations. Ihn et al. demonstrated that afatinib can suppress osteoclast differentiation induced by RANKL, reduce the expression of osteoclast‐specific markers, and attenuate bone resorption.^18^ An in vitro study showed that osimertinib can regress tumors, improve bone remodeling, and increase survival rates in mice with BoM models, suggesting its potential as a treatment for EGFR‐mutated NSCLC patients with BoM.^19^ A retrospective study of patients with metastatic NSCLC and EGFR mutations receiving first‐line EGFR–TKI treatment revealed that combining denosumab with EGFR–TKI leads to longer OS than EGFR–TKI monotherapy.^20^ However, another study found that denosumab did not impact progression‐free survival (PFS) or OS.^21^ Recent research has explored combining EGFR–TKIs with vascular endothelial growth factor (VEGF) inhibitors in treating NSCLC patients with EGFR mutations.^22^, ^23^ Some studies have observed high VEGF and receptor expression in BoM in breast or liver cancer.^24^, ^25^ Chen et al. reported that antiangiogenic therapy (AAT) might improve survival chances for EGFR‐mutated NSCLC patients with BoM.^7^

The findings underscore the importance of identifying treatment options for EGFR‐mutated NSCLC patients with BoM. Consequently, this retrospective study has two objectives: (1) to compare the clinical characteristics and prognosis of EGFR‐mutated NSCLC patients with and without initial BoM; and (2) to identify effective hybrid treatment strategies.

## 2 METHODS

### 2.1 Study design and patient population

This retrospective study was designed to investigate patients diagnosed with stage IV EGFR‐mutated lung adenocarcinoma who received first‐line EGFR–TKI therapy. The patient population was divided into two groups based on the presence or absence of bone metastases (BoM) at initial diagnosis, determined through bone magnetic resonance imaging (MRI), bone scan, or positron emission tomography (PET). To investigate whether denosumab improves prognosis, we further analyzed the baseline characteristics of patients who received denosumab and those who did not. Data were collected from patients treated at China Medical University Hospital between January 2014 and December 2020, excluding those with insufficient medical data, stage IIIB/IIIC disease, or patients who did not undergo re‐biopsy upon disease progression. The study population includes participants from our previous study,^7^ which is part of the current study. Informed consent was waived by the Institutional Review Board of China Medical University Hospital (CMUH 110‐REC3‐110). Baseline patient characteristics were recorded, including age, sex, smoking history, ECOG performance status, initial TNM staging, EGFR mutation subtype, metastasis patterns, first‐line treatment actions, the status of the EGFR T790M mutation at disease progression (T790M mutations were identified through tissue biopsy and/or liquid biopsy), denosumab and AAT administration, and subsequent osimertinib utilization following disease progression.

### 2.2 Antiangiogenesis therapy and denosumab treatment

Since bevacizumab and ramucirumab are not covered for lung cancer treatment under Taiwan's National Health Insurance program, we administered reduced dosages of these drugs in our study. Bevacizumab was given every 3 weeks at a dose of 7.5 mg/kg body weight, and ramucirumab was administered every 3 weeks at a dose ranging from 5 to 10 mg/kg body weight. Our final analysis included all participants who received a minimum of 3 cycles of combination therapy with either bevacizumab or ramucirumab. Patients in the denosumab group received a subcutaneous dose of 120 mg of denosumab once a month, starting within 1 month of BoM diagnosis and continuing for at least three treatments. During this period, they did not receive any other treatment for their BoM, including zoledronic acid.

### 2.3 Assessment of outcomes

In our previous research, we discussed methods for evaluating the treatment of patients.^7^ To assess the baseline stage of lung cancer, we employed various imaging techniques such as computed tomography (CT), brain imaging including brain CT and/or MRI, and PET. Tumor response monitoring was conducted every 3–4 months after initiating EGFR–TKI therapy, using chest CT and clinical judgment based on brain CT or MRI. Progression‐free survival (PFS) was defined as the period from the start of EGFR–TKI therapy until tumor progression, detected using the Response Evaluation Criteria in Solid Tumors v1.1, death, or censoring at the last follow‐up on November 30, 2022. Similarly, OS was defined as the time between the commencement of EGFR–TKI therapy and death or censoring at the last follow‐up on November 30, 2022.

### 2.4 Statistical analyses

Data analysis was conducted using MedCalc for Windows, version 18.10 (MedCalc Software, Ostend, Belgium). Continuous variables with normal distributions are presented as mean ± standard deviations, while those with non‐normal distributions are reported as medians and interquartile ranges. We used the *t*‐test to determine differences between groups for continuous data with normal distributions and the Kruskal–Wallis test for non‐normally distributed data. Categorical variables are expressed as numbers and percentages, with the chi‐squared test used for comparing differences between independent groups. The Kaplan–Meier method was employed to evaluate PFS and OS. Univariate and multivariate Cox proportional hazards regression analyses were performed to identify independent prognostic factors of OS. A *p*‐value <0.05 was considered statistically significant, and the strength of association was measured as a hazard ratio (HR) with an associated 95% confidence interval (CI).

## 3 RESULTS

**FIGURE 1:**
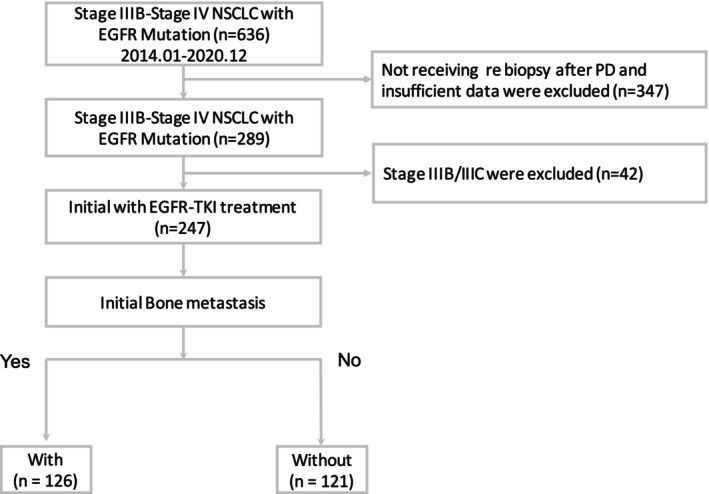
Flowchart showing patient selection. EGFR, epidermal growth factor receptor; NSCLC, non‐small‐cell lung cancer; PD, progressive disease; TKI, tyrosine kinase inhibitor.

Between January 2014 and December 2020, 636 patients were diagnosed with EGFR‐mutated stage IIIB–IV NSCLC, out of which 247 received EGFR–TKI as first‐line therapy and met the inclusion criteria. These patients were divided into two groups based on the presence or absence of BoM at initial diagnosis: 126 in the BoM group and 121 in the non‐BoM group (Figure 1). The baseline characteristics of the study subjects are presented in Table 1. Except for the pattern of metastasis, the demographic characteristics of the two groups were similar. Liver and adrenal metastases were more common in the BoM group than in the non‐BoM group (26.2% vs. 8.3%, *p* = 0.002; and 18.3% vs. 5.8%, *p* = 0.003, respectively). However, the non‐BoM group exhibited a higher incidence of pleural involvement compared to the BoM group (54.5% vs. 36.5%, *p* = 0.004). Notably, multi‐organ metastases and extrathoracic metastases involving three or more organs were more frequent in the BoM group (*p* < 0.001).

**TABLE 1:** The baseline clinical characteristics of *EGFR*‐mutated NSCLC patients with or without bone metastasis (BoM).

	Total (*n* = 247)	Bone metastasis (*n* = 126)	No bone metastasis (*n* = 121)	*p*‐value
Age, years (SD)	60.7 ± 0.7	59.7 ± 10.9	61.7 ± 11.0	0.162
Sex, male (%)	81 (32.8)	44 (34.9)	37 (30.6)	0.468
Smoking	54 (21.9)	26 (21.5)	28 (22.2)	1.000
ECOG PS ≥2	20 (8.1)	13 (10.3)	7 (5.8)	0.192
EGFR mutation				
Del 19	133 (53.8)	63 (50.0)	70 (57.9)	0.194
L858R	103 (41.7)	59 (46.8)	44 (36.4)	
Other	11 (4.5)	4 (3.2)	7 (5.8)	
Metastatic sites				
Lung to lung	123 (49.8)	56 (44.4)	67 (55.4)	0.086
Pleural	112 (45.3)	46 (36.5)	66 (54.5)	0.004
CNS	51 (20.6)	31 (24.6)	20 (16.5)	0.117
Liver	43 (17.4)	33 (26.2)	10 (8.3)	0.002
Adrenal gland	30 (12.1)	23 (18.3)	7 (5.8)	0.003
Other metastasis	45 (18.2)	26 (20.6)	19 (15.7)	0.316
Metastatic site ≥3	77 (31.2)	61 (48.3)	6 (13.2)	<0.001
Extra thoracic Mets ≥3	31 (12.5)	31 (24.6)	0 (0)	<0.001
EGFR‐TKI				
Gifitinib	74 (30.0)	35 (27.8)	39 (32.2)	0.662
Erlotonib	88 (35.6)	49 (38.9)	39 (32.2)	
Afatinib	82 (33.2)	41 (42.5)	41 (33.9)	
Anti‐angiogenetic drug	67 (27.1)	41 (32.5)	26 (21.5)	0.051
Osimertinib	128 (51.8)	67 (53.2)	61 (50.4)	0.665

Abbreviations: CNS, central nerve system; ECOG, Eastern Cooperative Oncology Group; EGFR, epithelial growth factor receptor; NSCLC, non‐small‐cell lung cancer.

**FIGURE 2:**
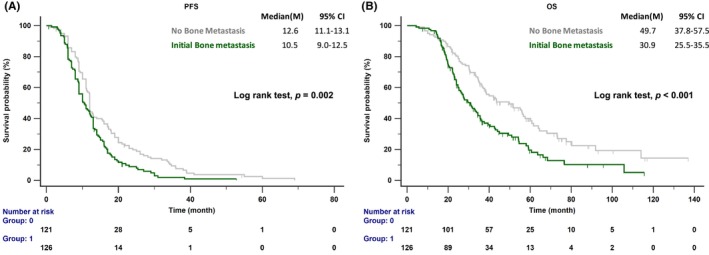
(A) PFS of patients treated for *EGFR*‐mutated NSCLC with or without bone metastasis (BoM). (B) OS of patients treated for *EGFR*‐mutated NSCLC with or without BoM. PFS, progression‐free survival; OS, overall survival.

**FIGURE 3:**
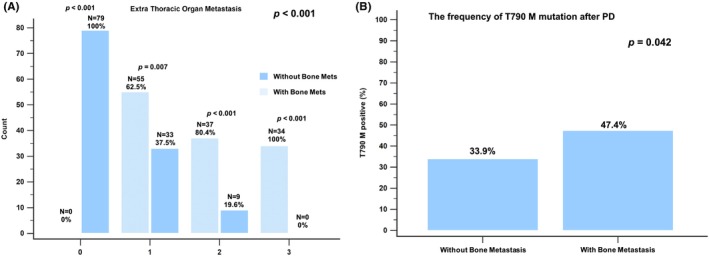
(A) The proportions of *EGFR*‐mutated NSCLC patients with or without bone metastasis (BoM) with extrathoracic metastasis. (B) The incidence of T790M in *EGFR*‐mutated NSCLC patients with or without BoM after 1st line treatment failure. EGFR, epidermal growth factor receptor; PD, progressive disease.

After a median follow‐up period of 67.4 months (range 62.2–75.7 months), 168 of the 247 patients had died. The BoM group experienced shorter PFS (10.5 months; 95% CI: 9.0–12.5 months) and OS (30.9 months; 95% CI: 25.5–35.5 months) compared to the non‐BoM group (PFS: 12.6 months, 95% CI: 11.1–13.1, *p* = 0.002; OS: 49.7 months, 95% CI: 37.8–57.5, *p* < 0.001; see Figure 2A,B). The BoM group had a higher incidence of extrathoracic metastases than the non‐BoM group. Among patients with extrathoracic organ metastasis, those with and without BoM accounted for 62.5% and 37.5% (*p* = 0.007) of the cases with one metastatic site, 80.4% and 19.6% (*p* < 0.001) of the cases with two metastatic sites, and 100% and 0% (*p* < 0.001) of the cases with three metastatic sites, respectively. This higher tumor burden in the BoM group might explain the more frequent opportunity for obtaining repeated biopsy specimens during clinical practice, possibly leading to the higher incidence of the T790M mutation compared to the non‐BoM group (47.4% vs. 33.9%, *p* = 0.042; see Figure 3A,B).

**FIGURE 4:**
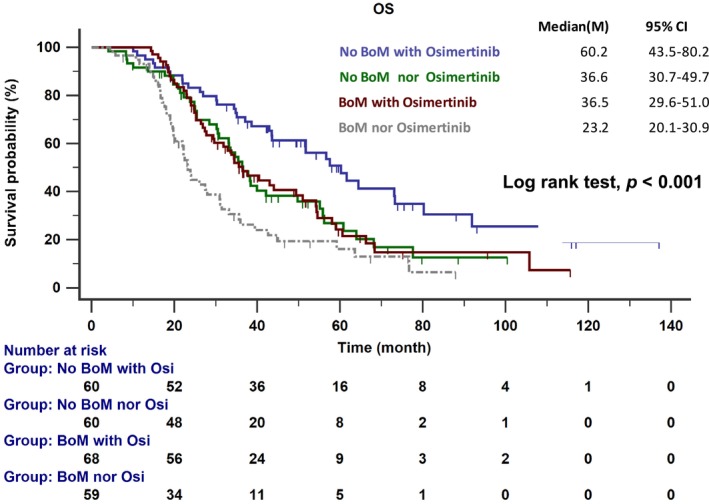
The overall survival (OS) of *EGFR*‐mutated NSCLC patients with or without bone metastasis who received osimertinib treatment and those who did not. EGFR, epidermal growth factor receptor; NSCLC, non‐small‐cell lung cancer.

To assess whether osimertinib could mitigate the negative impact of BoM, data on salvage therapy with osimertinib following initial treatment failure were analyzed. Among the patients who received subsequent osimertinib, 68 were in the BoM group and 60 in the non‐BoM group. The BoM patients treated with subsequent osimertinib exhibited not only longer OS compared to BoM patients who did not receive osimertinib (36.5 vs. 23.3 months; *p* < 0.001), but their OS rate was also similar to that of the non‐BoM patients who did not receive subsequent osimertinib (36.5 vs. 36.6 months; see Figure 4).

**FIGURE 5:**
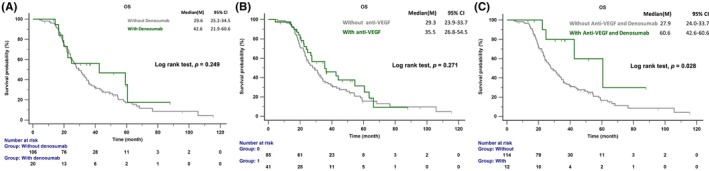
The overall survival of patients with *EGFR*‐mutated NSCLC treated with (A) denosumab; (B) antiangiogenesis therapy; (C) denosumab and antiangiogenesis therapy. EGFR, epidermal growth factor receptor; OS, overall survival.

This study also examined the benefits of AAT and denosumab for patients in the BoM group, which was further divided based on whether patients received denosumab. Twenty patients received denosumab, 41 received AAT, and 12 received both denosumab and AAT. The group treated with denosumab had a higher proportion of patients receiving AAT compared to the group without denosumab (60% vs. 27.4%; *p* = 0.004; Table 2). Patients who received both AAT and denosumab exhibited an increased OS rate compared to those who did not receive these treatments (60.6 vs. 27.9 months; *p* = 0.028; see Figure 5). Multivariate analysis was performed to test the prognostic factors identified as significant in univariate analyses, and it identified good performance status, subsequent osimertinib treatment, and combined AAT and denosumab treatment as independent positive prognostic factors for OS (Table 3).

**TABLE 2:** The baseline clinical characteristics of *EGFR*‐mutated NSCLC patients with bone metastasis (BoM) who received or did not receive denosumab.

	Total BoM (*n* = 126)	With denosumab (*n* = 20)	Without denosumab (*n* = 106)	*p*‐value
Age ≥ 65, years (%)	40 (31.7)	3 (15.0)	37 (34.9)	0.081
Sex, male (%)	44 (34.9)	6 (30.0)	38 (35.8)	0.616
Smoking (%)	28 (22.2)	1 (5.0)	27 (25.5)	0.044
ECOG PS ≥2 (%)	13 (10.3)	3 (15.0)	10 (9.4)	0.433
EGFR mutation (%)				
Del 19	63 (50.0)	6 (30.0)	57 (53.8)	0.043
L858R	59 (46.8)	12 (60.0)	47 (44.3)	
Other	4 (3.2)	2 (10.0)	2 (1.9)	
Metastatic sites (%)				
Lung to lung	56 (44.4)	9 (45.0)	47 (44.3)	0.956
Pleural	46 (36.5)	8 (40.0)	38 (35.8)	0.725
CNS	31 (24.6)	4 (20.0)	27 (25.5)	0.604
Liver	33 (26.2)	3 (15.0)	30 (28.3)	0.216
Adrenal gland	23 (18.3)	2 (10.0)	21 (19.8)	0.299
Other metastasis	26 (20.6)	6 (30.0)	20 (18.9)	0.261
Metastatic site ≥3	61 (48.4)	8 (40.0)	53 (50.0)	0.414
Extra thoracic Mets ≥3	33 (26.2)	4 (20.0)	29 (27.4)	0.494
EGFR‐TKI (%)				
Gifitinib	35 (28.0)	3 (15.0)	32 (30.5)	0.365
Erlotonib	39 (39.2)	9 (45.0)	40 (38.1)	
Afatinib	41 (32.8)	8 (40.0)	33 (31.4)	
Anti‐angiogenetic drug (%)	41 (32.5)	12 (60.0)	29 (27.4)	0.004
Osimertinib (%)	67 (53.2)	10 (50.0)	57 (53.8)	0.757

Abbreviations: CNS, central nerve system; ECOG, Eastern Cooperative Oncology Group; EGFR, epithelial growth factor receptor; NSCLC, non‐small‐cell lung cancer.

**TABLE 3:** Cox proportional hazards regression analysis of the OS of patients with *EGFR*‐mutant NSCLC with bone metastasis (BoM).

Clinical factors	Univariate		Multivariate	
	OR (95% CI)	*p*‐value	OR (95% CI)	*p*‐value
Age ≥ 65	1.20 (0.77–1.87)	0.416	–	–
ECOG ≥2	2.44 (2.48–4.67)	0.014	2.86 (1.48–5.53)	0.002
Osimertinib	0.14 (0.39–0.90)	0.015	0.59 (0.39–0.89)	0.012
Denosumab	0.69 (0.36–1.30)	0.231	1.30 (0.58–2.90)	0.517
Anti‐VEGF	0.77 (0.48–1.22)	0.263	1.05 (0.63–1.74)	0.857
Anti‐VEGF and denosumab	0.34 (0.13–0.93)	0.013	0.23 (0.06–0.87)	0.031

Abbreviations: ECOG, Eastern Cooperative Oncology Group; EGFR, epithelial growth factor receptor; NSCLC, non‐small‐cell lung cancer;VEGF, vascular epithelial growth factor.

## 4 DISCUSSION

Our findings reveal that EGFR‐mutated NSCLC patients with BoM have shorter PFS and OS compared to those without BoM. Additionally, patients with BoM are more likely to develop metastases in multiple organs. After initial treatment failure, osimertinib appears to be a preferable choice for sequential therapy, especially given the high incidence of the T790M mutation in EGFR‐mutated NSCLC patients with BoM. The triple combination therapy of EGFR–TKI, denosumab, and an anti‐VEGF agent can synergistically improve OS in these patients. To our knowledge, our study is the first to investigate the survival benefits of combining AAT and denosumab for EGFR‐mutated NSCLC patients with BoM.

Several previous studies have established that BoM is linked to a poor prognosis in EGFR‐mutated NSCLC patients.^5^, ^8^, ^9^ Our study similarly found that EGFR‐mutated NSCLC patients with BoM experience shorter PFS (10.5 vs. 12.6 months, *p* = 0.002, Figure 2A) and OS (30.9 vs. 49.7 months, *p* < 0.001) compared to those without. BoM is associated with multi‐organ metastases, including brain and liver metastases, which are known factors of poor prognosis.^26^, ^27^ The recent SEER database study indicated that lung cancer prognosis worsens with an increase in the number of metastatic sites.^28^ In bone metastatic cancer, the RANK/RANKL pathway plays a vital role in bone metabolism, immunity, and tumorigenesis.^29^ Its expression has been identified as a negative prognostic factor in breast cancer patients.^14^ The RANKL/RANK pathway is also a critical immune mediator. Recent studies have connected anti‐RANKL therapy with improved responses to immunotherapy in melanoma, NSCLC, and renal cell carcinoma.^30^ RANKL is a negative prognostic indicator in patients with advanced KRAS‐mutated lung adenocarcinoma.^31^ Furthermore, the RANKL/RANK pathway contributes to angiogenesis and enhances vascular permeability via RANK‐expressing endothelial cells, potentially impacting extravasation and metastasis processes, leading to greater tumor burden.^32^ Patients with BoM are more likely to develop multi‐organ metastasis. Thus, we hypothesize that the RANK/RANKL pathway may be associated with poor prognosis in NSCLC patients with BoM.

Denosumab has been demonstrated to have anti‐tumor effects by blocking the interaction between RANK and RANKL.^9^, ^11^, ^33^ This inhibition suppresses the nuclear factor‐kappa B pathway, which is related to lung carcinogenesis, and enhances intercellular adhesion molecule one expression and the MEK/extracellular signal‐regulated kinase pathway, subsequently promoting tumor cell migration.^12^ The combination of denosumab and EGFR–TKI has been found to increase OS in EGFR‐mutated NSCLC patients with BoM. In a retrospective study of 190 NSCLC patients with BoM, combining denosumab with first‐ or second‐generation EGFR–TKI was associated with improved OS (26.6 vs. 20.1 months; *p* = 0.015).^20^ However, another real‐world study reported that denosumab had no significant prognostic effects in lung adenocarcinoma patients with BoM treated with first‐line EGFR–TKIs.^21^ In our study, combining EGFR–TKI with denosumab showed a non‐significant trend toward improving the OS of EGFR‐mutated NSCLC patients with BoM. These inconsistent results could be attributed to factors such as insufficient sample size, variation in additional treatments at different institutions, and limited follow‐up duration.

An animal study on osteoclasts demonstrated that the ERK1/2 pathway regulates osteoclast invasion induced by RANKL and VEGF.^34^ VEGF is involved in regulating RANK/RANKL‐mediated osteoclastogenesis.^35^ The immune modulatory effects of antiangiogenic agents targeting the VEGF/VEGF receptor on various immune cells, including effector T cells, Tregs, myeloid‐derived suppressor cells, dendritic cells, tumor‐associated macrophages (TAMs), and mast cells, in the tumor microenvironment of NSCLC, have been well‐documented.^36^ Additionally, the RANK/RANKL pathway is linked to resistance to immunotherapy, as RANKL attracts TAMs and RANKL/RANK signaling in M2 macrophages promotes Treg lymphocyte proliferation, creating an immunosuppressive environment.^37^ There is potential crosstalk between the RANK/RANKL and VEGF pathways in the metastatic bone and tumor microenvironment of NSCLC. A systematic review and meta‐analysis showed that combining EGFR TK inhibitors and anti‐VEGF agents in patients with EGFR‐mutated NSCLC could improve PFS.^38^ Our previous study indicated that EGFR‐mutated NSCLC patients with BoM could benefit in survival by receiving additional anti‐VEGF treatment.^7^ In the current study, we found that the combination therapy of EGFR–TKI, denosumab, and an anti‐VEGF agent significantly improved OS compared to EGFR–TKI monotherapy in patients with EGFR‐mutated NSCLC. The combination therapy group had a longer OS of 60.6 months versus 27.9 months in the monotherapy group (*p* = 0.028). Moreover, after multivariate analysis, the combination of denosumab and an anti‐VEGF agent was identified as a prognostic factor, with an odds ratio of 0.23 (95% CI: 0.06–0.87, *p* = 0.031).

Osimertinib, a third‐generation EGFR–TKI, is extensively used to treat NSCLCs that harbor the T790M mutation and is an effective sequential therapy for EGFR‐mutated NSCLC patients with an acquired T790M mutation after first‐ or second‐generation EGFR–TKI treatment.^39^ It is also preferred as a first‐line therapy over first‐generation EGFR–TKI drugs.^40^ A retrospective study involving advanced NSCLC patients receiving gefitinib/erlotinib (*n* = 183), osimertinib (*n* = 150), or afatinib (*n* = 55) found that osimertinib significantly prolonged PFS in patients with BoM and the exon 19 deletion, compared to other EGFR–TKIs.^41^ An in vitro study showed that osimertinib induced tumor regression, improved survival in mice, and caused bone remodeling in bone metastatic models.^19^ The present study identified sequential therapy with osimertinib as a prognostic factor for EGFR‐mutated NSCLC patients with BoM. Our findings indicate that EGFR‐mutated NSCLC patients with BoM receiving first‐ or second‐generation EGFR–TKI are more likely to develop T790M resistance after TKI failure (47.4% vs. 33.9%, *p* = 0.042). This could be another factor contributing to the improved OS observed with osimertinib sequential therapy.

The present study has several limitations. First, its retrospective design includes only a limited number of patients who received denosumab or anti‐VEGF treatment. Despite denosumab being commonly used in Taiwan following a reimbursement program initiated by National Health Insurance on December 1, 2015, not all physicians prescribe it for managing bone diseases or skeletal‐related events (SREs) in NSCLC patients.^6^, ^20^, ^21^ Additionally, anti‐VEGF medicine is not covered by Taiwan Health Insurance. Therefore, large‐scale clinical trials are necessary to conclusively demonstrate the benefits of combining denosumab and anti‐VEGF with EGFR–TKIs. Second, in real‐world scenarios, the start time for denosumab varies, complicating the assessment of its impact on PFS. In our study, OS was the primary indicator when combining denosumab with EGFR–TKI treatment. Third, our study does not report on SREs, which previous studies have identified as a poor prognostic factor.^20^, ^21^ Fourth, we used different dosages of bevacizumab (7.5 mg/kg every 3 weeks) and intravenous ramucirumab (5–10 mg/kg every 3 weeks) compared to prior trials,^22^, ^23^ due to these drugs not being covered for lung cancer treatment under Taiwan's National Health Insurance, leading to the use of reduced dosages. Previous research showed that combining chemotherapy with 7.5 mg/kg of bevacizumab was as effective as using 15 mg/kg.^42^ Finally, this study mainly involved patients receiving first or second‐generation EGFR–TKIs as first‐line therapy. The global shift to using osimertinib as a first‐line treatment may influence our findings. However, the optimal sequencing of these TKIs is crucial for survival outcomes. Given the limited treatment options post‐osimertinib, subsequent osimertinib therapy after first or second‐generation EGFR–TKI failure remains a feasible strategy. Despite these limitations, our study is the first to explore combination therapy with denosumab and AAT for EGFR‐mutated NSCLC patients with BoM.

## 5 CONCLUSION

Our study indicates that BoM is common in EGFR‐mutated NSCLC patients and is associated with a poor prognosis. For those experiencing EGFR–TKI failure and having BoM, osimertinib is a preferred option for sequential therapy due to the high incidence of the T790M mutation. The combination of an EGFR–TKI with either denosumab, an anti‐VEGF agent, or both, has been shown to improve OS in these patients. Specifically, the hybrid combination of EGFR–TKI, denosumab, and an anti‐VEGF agent appears to have a synergistic effect on OS. Further research is necessary to confirm the synergistic impact of combining denosumab and anti‐VEGF agents.

## AUTHOR CONTRIBUTIONS

**Wei‐Chun Chen:** Conceptualization (equal); data curation (equal); formal analysis (equal); funding acquisition (equal); investigation (equal); methodology (equal); project administration (equal); resources (supporting); supervision (equal); validation (equal); visualization (equal); writing – original draft (equal); writing – review and editing (equal). **Wen‐Chien Cheng:** Conceptualization (lead); data curation (lead); formal analysis (lead); investigation (lead); methodology (lead); resources (supporting); software (lead); supervision (lead); validation (lead); visualization (lead); writing – original draft (lead); writing – review and editing (lead). **Chieh‐Lung Chen:** Conceptualization (supporting); data curation (equal); formal analysis (supporting); validation (supporting); visualization (supporting). **Wei‐Chih Liao:** Data curation (supporting); resources (supporting); supervision (supporting); validation (supporting). **Chia‐Hung Chen:** Data curation (supporting); formal analysis (supporting); methodology (supporting); resources (supporting); supervision (supporting); validation (supporting); visualization (supporting). **Hung‐Jen Chen:** Conceptualization (equal); data curation (equal); investigation (equal); resources (supporting); validation (supporting); visualization (supporting). **Chih‐Yen Tu:** Data curation (equal); funding acquisition (equal); project administration (equal); resources (equal); supervision (equal); validation (equal); visualization (equal). **Chi‐Chien Lin:** Conceptualization (equal); formal analysis (equal); funding acquisition (equal); investigation (equal); methodology (equal); project administration (equal); software (equal); supervision (equal); validation (equal); visualization (equal); writing – original draft (equal); writing – review and editing (equal). **Te‐Chun Hsia:** Conceptualization (equal); data curation (equal); formal analysis (equal); funding acquisition (equal); investigation (equal); methodology (equal); project administration (equal); resources (equal); supervision (equal); validation (equal); visualization (equal).

## CONFLICT OF INTEREST STATEMENT

The authors declare that they have no conflicts of interest.

## ETHICS STATEMENT

The Institutional Review Board of China Medical University Hospital (CMUH110‐REC1‐244) approved this retrospective study in compliance with the ethical standards of the Declaration of Helsinki. The requirement for individual patient consent was waived by the Ethics Review Board because of the retrospective study design.

## Data Availability

All data generated or analyzed in this study are included in this published article.
